# Characterization and Expression Analysis of a Fiber Differentially Expressed Fasciclin-like Arabinogalactan Protein Gene in Sea Island Cotton Fibers

**DOI:** 10.1371/journal.pone.0070185

**Published:** 2013-07-17

**Authors:** Hengwei Liu, Ruifeng Shi, Xingfen Wang, Yuxin Pan, Zhikun Li, Xinlei Yang, Guiyin Zhang, Zhiying Ma

**Affiliations:** 1 North China Key Laboratory for Crop Germplasm Resources of Education Ministry, Agricultural University of Hebei, Baoding, China; 2 School of Chemistry and Biological Engineering, Suzhou University of Science and Technology, Suzhou, China; Kyushu Institute of Technology, Japan

## Abstract

Fasciclin-like arabinogalactan (FLA) protein is a cell-wall-associated protein playing crucial roles in regulating plant growth and development, and it was characterized in different plants including Upland cotton (*Gossypium hirsutum* L.). In cDNA-AFLP analysis of 25 DPA (days post anthesis) fiber mRNA, two FLA gene-related transcripts exhibit differential expression between Sea Island cotton ( *G* *. barbadense* L.) and Upland cotton. Based on the transcript-derived fragment, RACE-PCR and realtime PCR technique, *GbFLA5* full-length cDNA was isolated and its expression profiles were characterized in both cotton plant tissues and secondary cell wall (SCW) fibers in this study. The 1154 bp *GbFLA5* cDNA contains an ORF of 720 bp, encoding GbFLA5 protein of 239 amino acids residues in length with an estimated molecular mass of 25.41 kDa and isoelectric point of 8.63. The deduced GbFLA5 protein contains an N-terminal signal sequence, two AGP-like domains, a single fasciclin-like domain, and a GPI anchor signal sequence. Phylogenetic analysis shows that GbFLA5 protein is homologous to some known SCW-specific expressed FLAs of plant developing xylem, tension wood and cotton fibers. In the SCW deposition stage from 15 to 45 DPA detected, *FLA5* maintains a significantly higher expression level in Sea Island cotton fibers than in Upland cotton fibers. The increasing *FLA5* transcript abundance coincided with the SCW deposition process and the expression intensity differences coincided with their fiber strength differences between Sea Island cotton and Upland cotton. These expression profile features of *GbFLA5* in cotton fibers revealed its tissue-specific and SCW developmental stage-specific expression characters. Further analysis suggested that GbFLA5 is a crucial SCW-specific protein which may contribute to fiber strength by affecting cellulose synthesis and microfibril deposition orientation.

## Introduction

Cotton fiber is the most important raw materials of spinning industry worldwide. The developing spinning technology and the increasing desire for better cloth quality demand the fiber qualities, especially fiber strength to be constantly improved. Sea Island cotton ( *Gossypium* *barbadense* L.) is highly valued for its superior fiber qualities, especially fiber strength for improving Upland cotton (*Gossypium hirsutum* L.) in breeding efforts. However, the negative correlations among fiber quality parameters and lint yield [1,2], the genetic instability and the unwanted agronomic characters of wide-hybridization progeny make it difficult to improve the Upland cotton fiber strength via interspecies crossing with the Sea Island cotton. Therefore identification and isolation of key genes and their regulating elements, as well as elucidating their functional mechanism caught cotton breeders’ great interests.

Cotton fiber qualities are determined by cotton fiber development process, which is generally classified into four clearly characterized, but overlapping stages, including fiber initiation (−3 to 5 days post anthesis, DPA), elongation (5 to 25 DPA), secondary cell wall (SCW) deposition (15 to 45 DPA) and maturation/dehydration (45 DPA and thereafter) [3]. During SCW stage, cellulose microfibrils deposit on cell wall which enables fibers to be an excellent mechanical structure with enough strength and flexibility. It was considered that the development of SCW is crucial to the final fiber qualities, especially for fiber strength and micronaire value [4]. The different fiber qualities of Sea Island cotton and Upland cotton, therefore raise the possibility of differential gene expression of these two *Gossypium* species during SCW deposition stage. We then focus on genes specially expressed in SCW fibers which could potentially be valuable genetic materials (genes and strains) in breeding.

Meanwhile, cotton fiber is an excellent model for studying cell differentiation, cell polar elongation and cell wall biosynthesis [5]. Exploring the differentially expressed genes between the two *Gossypium* species with different fiber characters can give valuable insights into the molecular mechanisms for plant cell polar elongation and SCW thickening.

In our previous studies [6,7], using cDNA-AFLP technology, transcriptome analyses were employed to reveal differentially SCW-expressed genes between two *Gossypium* species, the Sea Island cotton and the Upland cotton. A set of species-specific transcript-derived fragments (TDFs) were obtained. Among them, two *Gossypium* *barbadense* -originated TDFs that are both homologous to a putative fasciclin-like arabinogalactan protein (FLA) were detected differentially expressed in two independent studies with different restriction enzyme combinations in the cDNA-AFLP analysis. Therefore it indicated a correlation of a SCW-expressed FLA gene with the process of affecting the SCW development difference between the two *Gossypium* species.

FLA protein belongs to a group of glycosylphosphatidylinositol (GPI)-anchored arabinogalactan proteins (AGPs). Existing evidences support multiple roles for AGPs in regulation of various processes associated with plant growth and development, including xylem differentiation [8,9], cell wall pectin plasticizer [10], cell expansion [11,12], shoot regeneration [13], root and pollen tube growth [14], stem biomechanics and cell wall architecture [15].

Fasciclin domains are 110 to 150 amino acid residues in length and with low sequence similarity. Proteins with fasciclin domains were first identified as an adhesion factor in *Drosophila* [16] and have since been isolated from algae [17], lichens [18], chick [19] and some higher plants [20]. AGP protein gene family in model plants of rice and *Arabidopsis* were widely investigated [21,22].

Identification of cotton AGP gene was first reported by Ji et al. [23]. Thereafter Liu et al. [24] and Huang et al. [25] discovered more FLAs or AGPs in Upland cotton. Nineteen *GhFLAs* were isolated from Upland cotton by Huang et al. [25] and Real-time PCR analysis revealed their regulated expression patterns both in different plant tissues and in developing fibers. For instance, *GhFLA1* and *GhFLA4* have their highest transcribing activities at 10 DPA while *GhFLA2* and *GhFLA6* at 20 DPA contrastively. These evidences implicated that the expression modulation of FLA proteins in regulating developing cotton fibers is diversified.

Up to the present, no *FLA* gene has been isolated from Sea Island cotton. Based on our previous cDNA-AFLP analysis, we sequenced the differentially expressed TDFs and isolated a *FLA* gene (designed as *GbFLA5*) and the expression pattern was further explored in this paper. We found that GbFLA5 protein contains a single FLA domain and falls into a subgroup with known SCW-specific expressed FLAs of plant developing xylem [26], tension wood [27] and cotton fibers [25] in phylogenetic analysis. Real-time PCR analysis indicated that the increasing *GbFLA5* transcript abundance coincided with the SCW deposition process and the expression intensity differences coincided with their fiber strength differences between Sea Island cotton and Upland cotton. These evidences suggested the correlation of the gene expression with fiber quality parameters.

## Materials and Methods

### Plant materials

Sea Island cotton cv. Pima 90-53 ( *G* *. barbadense* ) and Upland cotton cv. CRI 8 (*G. hirsutum*) were grown in a breeding nursery. Days were tagged on cotton bolls to determine the developing stages by days post anthesis (DPA). Fibers (10 DPA and thereafter) were collected from cotton bolls carefully at different stages. The other tissues (roots, hypocotyls and leaves) were obtained from cotton plants grown in the field. All the collected materials were frozen and grinded in liquid nitrogen immediately. The fine powders were stored at −80°C before DNA/RNA extraction.

### Preparation of genomic DNA, total RNA and single-strand cDNA

Genomic DNA was extracted from cotton leaves according to the protocol by Paterson et al. [28]. Total RNA was extracted from fibers and other tissues using PlantRNA reagent (Tiangen, China), according to the manufacturer’s protocol. After purification, DNA/RNA was quantitated to estimate their quality, purity and concentration by A260 measurement using the NanoDrop ND-1000 Spectrophotometer (NanoDrop Technologies, USA). The DNA/RNA quality was also confirmed by visualizing on agarose gel. Figure S1 is the visualized fiber RNA in this study.

Total RNA (pre-treated with DNase I) and Oligo(dT)_18_ was used to synthesize single-strand cDNA by the PrimeScript Reverse Transcriptase (Takara Bio, China), and the single-strand cDNA was used in the amplification of *GbFLA5* gene ORF and the expression analyses.

### cDNA-AFLP analysis and isolation of differentially expressed TDFs

Primer sequences, adaptor sequences and cDNA-AFLP analysis procedures were as described previously [6,7]. Two combinations of restriction enzyme of *Mse*I/*Eco*RI and *Apo*I/*Taq*I (New England Biolabs, USA) were used in cDNA-AFLP analysis. After selective amplification, the samples were denatured (95°C) and electrophoresed on 6.0% (w/v) denaturing polyacrylamide gel, then visualized by silver staining.

Isolation, amplification and sequencing of the differentially expressed TDFs (bands) of the Sea Island cotton and the Upland cotton were carried out as described previously [6]. The target bands were excised, extracted and used as template in amplification with the same primers as those used in the cDNA-AFLP analysis. The amplification products were electrophoresed on a 2.0% (w/v) agarose gel to check the fragments fidelity. The fragments were cloned using the pGEM-T clone Kit (Tiangen, China). At least three positive clones from each of the amplified fragments were sequenced by Shanghai Sangon Biological Engineering Technology and Service Company, China.

### Isolation and structural characterization of the *GbFLA5* full-length cDNA

Gene-specific primers of 5GSP and 3GSP were designed (Table 1) for rapid amplification of cDNA end (RACE) analysis to obtain the full-length sequence of the gene cDNA sequence. RACE amplifications were carried out using the Clontech SMART™ RACE cDNA amplification Kit (Clontech Laboratories Inc., USA) according to the manufacturer’s instruction.

**Table 1:** Oligonucleotide sequence of primers and their descriptions.

Primers	Sequence (5′ to 3′)	Description
ORFF	CCAAAACCCCTTCAACAATG	ORF forward primer
ORFR	AGGTCCCCGAATTTTCCAT	ORF reverse primer
3GSP	GCTGGTGCTATTTCACGTCGTCCCA	3’-RACE specific primer
5GSP	GAGAGGAAACTCACCGTCGCCGCT	5’-RACE specific primer
SpecificF	GCTTCGAGCCTTGCCATG	Specific forward primer
SpecificR	TGCACGCACAAATCACAAC	Specific reverse primer
EF1αF	GGTGGGATACAACCCTGACA	Forward primer of the internal control
EF1αR	TTGGGCTCATTGATCTGGTC	Reverse primer of the internal control

The RACE PCR reaction was performed for five cycles for 30 sec at 94°C, 30 sec at 70°C and 2 min at 72°C, followed by 25 cycles for 30 sec at 94°C, 30 sec at 68°C, 1 min at 72°C, and finally held at 72°C for an additional 5 min. The PCR products were purified, and were cloned into the pGEM-T vector and sequenced according to the methods mentioned above.

According to the assembled full-length cDNA sequence from RACE amplification, a couple of primers of ORFF and ORFR (Table 1) was designed and the PCR reaction was carried out to amplify the *GbFLA5* ORF. The PCR reaction was carried out with 50 ng of template DNA or cDNA, 0.625 U of *Pfu* DNA polymerase (MBI Fermentas, Uthuania), 10 mM of Tris-HCl (pH 8.4), 1.5 mM of MgCl_2_, 50 mM of KCl, 0.20 mM of each dNTP and 0.5 µM of each primer in a volume of 20 µL. Amplification conditions were as follows: predenaturation at 94° C for 1 min, followed by 25 cycles of amplification (45 sec at 94° C, 45 sec at 58° C, 3 min at 72° C) and by 10 min at 72° C. The purified PCR products were inserted into the pGEM-T vector (Tiangen, China) and completely sequenced according to the methods mentioned above. Comparing the ORF sequences between DNA and cDNA was preformed to predict the possible introns.

### Bioinformatic analysis, sequence alignment and phylogenetic analysis

Sequence encoding GbFLA5 was determined by homology searches in the NCBI databases using the BLAST program (http://www.ncbi.nlm.nih.gov/BLAST). DNAstar software was employed to assemble a full-length cDNA sequence from the two original TDF sequences, 5′ RACE and 3′ RACE overlapping sequences. Online ORF Finder (http://www.ncbi.nlm.nih.gov/gorf/gorf.html) was used to search gene ORF and translated into the corresponding amino acid sequence. Basic properties of isoelectric point and molecular weight of the predicted protein of GbFLA5 were estimated by using DNAstar software. Conserved domains of the predicted GbFLA5 were recognized through searching in the Pfam database using online SMART program (http://smart.embl-heidelberg.de/). A signal peptide sequence of the predicted GbFLA5 was also identified using Signal 3.0 Server (http://www.cbs.dtu.dk/services/SignalP-3.0/). Similarly, the putative GPI anchor signal in GbFLA5 was revealed using the online program analysis of big-PI Plant Predictor (http://mendel.imp.ac.at/sat/gpi/gpi_server.html). Multiple sequence alignments were conducted using the program ClustalW2 (http://www.ebi.ac.uk/Tools/msa/clustalw2) and the produced alignment were manually edited. Phylogenetic analysis and statistical neighbour-joining bootstrap tests of the phylogenies were performed with the Mega 4.0 package by using the bootstrap method with 1000 bootstrap iterations [29].

### Expression profile of *FLA5* transcript level in cotton

Semi-quantitative RT-PCR was performed with a suitable amount of diluted cDNA to investigate tissue-specific *FLA5* mRNA content in different cotton plant organs including leaf, root, hypocotyl and 25 DPA fiber. The 25 DPA fiber was used as a representative fiber in SCW stages. To standardize the results, the relative abundance of *EF1α* was also determined and used as internal control. The primer combinations of SpecificF and SpecificR for semi-quantitative RT-PCR were described in Table 1. The semi-quantitative RT-PCR for *FLA5* and *EF1α* was performed at the same conditions as following: denaturation for 1 min at 94° C, followed by 25 cycles of 30s at 94° C, 30s at 56° C and 30s at 72° C, and with a final extension for 5 min at 72° C. Finally, 10 µL PCR products were separated on 2% (w/v) agarose gel for visualization.

Real-time quantitative PCR was carried out to explore the expression patterns of *FLA5* in developing cotton fibers. The assay was carried out on LightCycler 1.5 Machine (Roche Diagnostics Corporation, Switzerland) with SYBR Green I fluorescence dye (Takara Bio, China). The amplifications were performed with a 20 µL reaction system containing 10 µL of 2×SYBR *Premix Ex Taq*™, 0.5 µL each of 10 µM SpecificF and SpecificR (Table 1) primers and a suitable amount of diluted cDNA. The thermal profile for real-time PCR was pre-incubation for 30 sec at 95°C, followed by 45 cycles of 5 sec at 95°C, 10 sec at 56°C and 10 sec at 72°C. Subsequently, a melting curve analysis was run for one cycle of 0 sec at 95°C, 15 sec at 65°C and with 0 second at 95°C holding in the continuous acquisition mode, followed for 10 sec at 40°C as a final cooling step. Housekeeping gene of cotton *EF1α* was co-amplified as the internal standard. The standard curves for *FLA5* and *EF1α* were generated by running reactions of 10-fold dilution series (10 different cDNA concentrations). The relative standard curve method was used for the calculation of fold changes in gene expression [30]. Amplification specificity was evaluated by including a negative control without template presenting in parallel with each analysis. Fibers from the first or second boll node on the fifth to the eighth fruit branches from three plants were used as three biological replicates in the analysis. And the melting curves of the replicates are showed in Figure S2 and Figure S3.

## Results

### Differentially expressed *GbFLA5* in Sea Island cotton fibers revealed by cDNA-AFLP analysis

**Figure 1:**
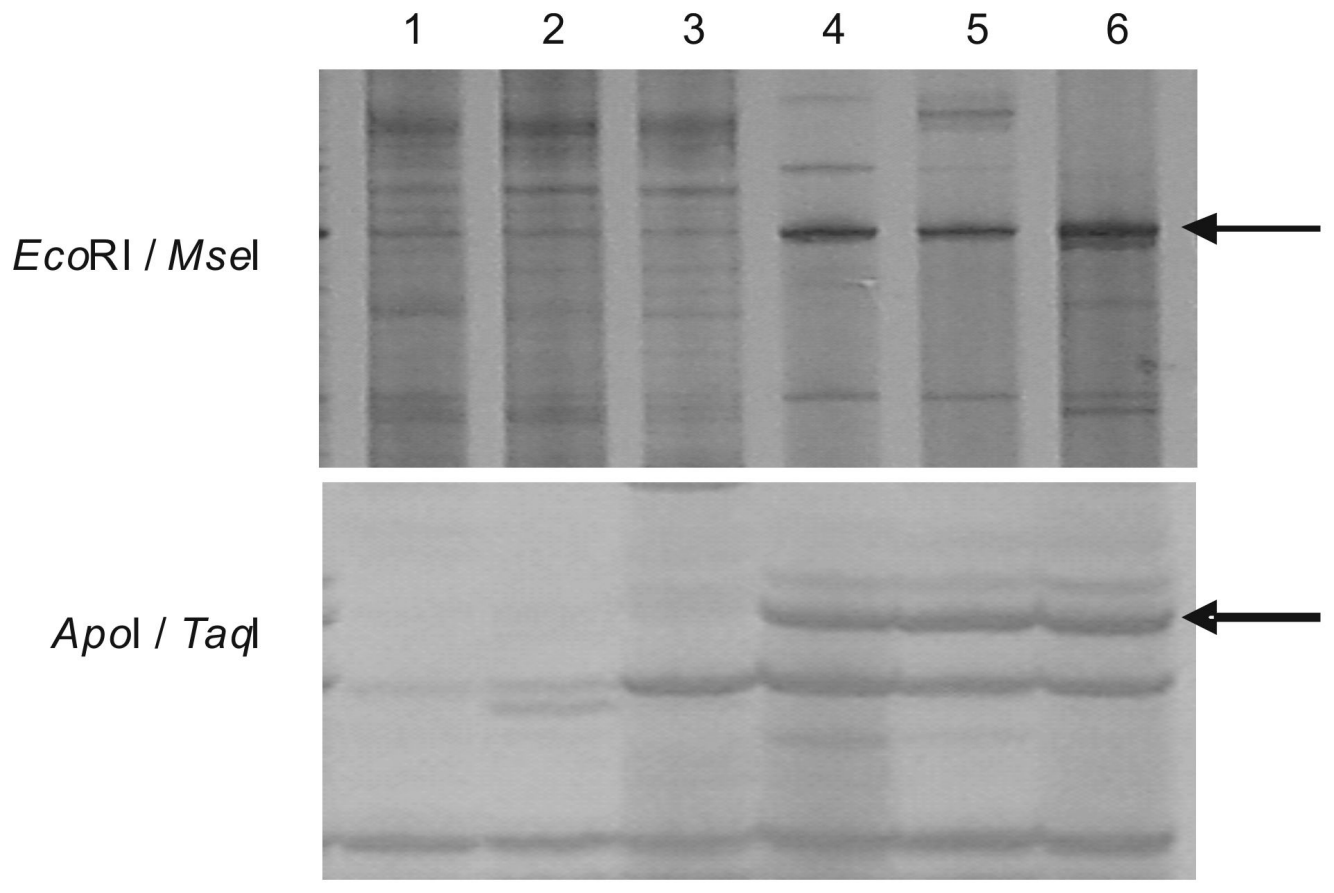
Visualized differentially expressed TDFs between Sea Island cotton fibers and Upland cotton fibers in cDNA-AFLP analyses. Lane 1 to 3 show Upland cotton (*G. hirsutum* cv. CRI 8) fibers and Lane 4 to 6 show Sea Island cotton ( *G* *. barbadense* cv. Pima90-53) fibers. *Eco*RI/*Mse*I and *Apo*I/*Taq*I are the restriction enzyme combinations used in cDNA-AFLP analyses. Arrowheads show the differentially expressed *FLA* TDFs.

Using restriction enzyme combinations of *Eco*RI/*Mse*I and *Apo*I/*Taq*I, the cDNA-AFLP analyses for fiber mRNAs were carried out in two consecutive years. The visualized differentially expressed TDF fingerprint was shown in Figure 1. The two TDFs, derived from combinations of *Eco*RI/*Mse*I and *Apo*I/*Taq*I, correspond to the 204 bp and 272 bp bands, respectively (including AFLP primer sizes). Comparison of the band intensity showed a dominant higher expression level in Sea Island cotton. The nearly indiscernible band in Upland cotton fibers indicated the infrequent transcripts in its SCW stage. Subsequently, the two differentially expressed TDFs were cloned, sequenced and spliced together. Database Blastx searching revealed that the spliced TDF displayed sequence homology to plant *FLA* genes in Genbank.

### Cloning of *GbFLA5* gene from Sea Island cotton

To obtain full-length *GbFLA5* cDNA with the original spliced TDF sequence, 5’- RACE and 3’- RACE amplifications were carried out and it was elongated to a full cDNA sequence of 1154 bp in length. Blastx analysis revealed a high identity with some plant FLA proteins in fasciclin superfamily. ORF finder predicted a 720 bp ORF by an initiation codon ATG and an in-frame stop codon TGA. The 5’- and 3’-UTR were also defined as 54 bp and 380 bp, respectively.

**Figure 2:**
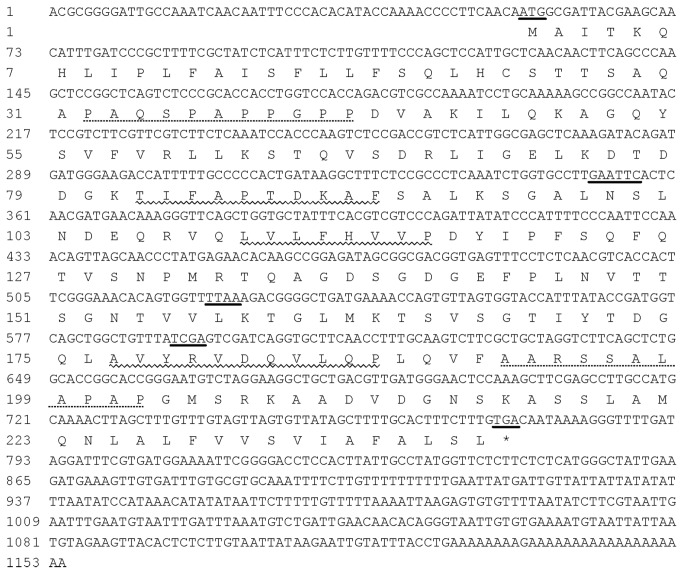
Full length cDNA sequence of *GbFLA5* and its deduced amino acid residues. Nucleotides underlined indicate the start codon of “ATG”, the effectively restriction site in cDNA-AFLP analysis (GAATTC for *Eco*RI and *Apo*I, TTAA for *Mse*I and TCGA for *Taq*I), and the stop codon of “TGA”, respectively; three conserved regions (H1, H2 and [YA] H) of smart00554 motif were ordinal characterized by wavy line; AGP-like domains are shown by the dashed line.

Despite sequence divergence in the 5’- and 3’-UTR region, the cloned ORF sequence share high similarity with *GhFLA5*（EF672631）and *GhAGP4*（EF470295）in Upland cotton. Therefore, the corresponding ORF identity of the two species was subject to further analysis, the Sea Island FLA gene presented here was designed as *GbFLA5*. The sequence of *GbFLA5* can be found in the GenBank database under accession number KC412182. Sequence conservation of the *GhFLA5* and *GbFLA5* ORFs indicated that the expression polymorphism of them revealed by cDNA-AFLP analysis could be due to their different upstream cis-element sequences or different trans-regulation factors. The full-sequence of *GbFLA5* cDNA and its deduced amino acid sequences were shown in Figure 2. The restriction sites used in the cDNA-AFLP analysis can be found in the sequence.

**Figure 3:**
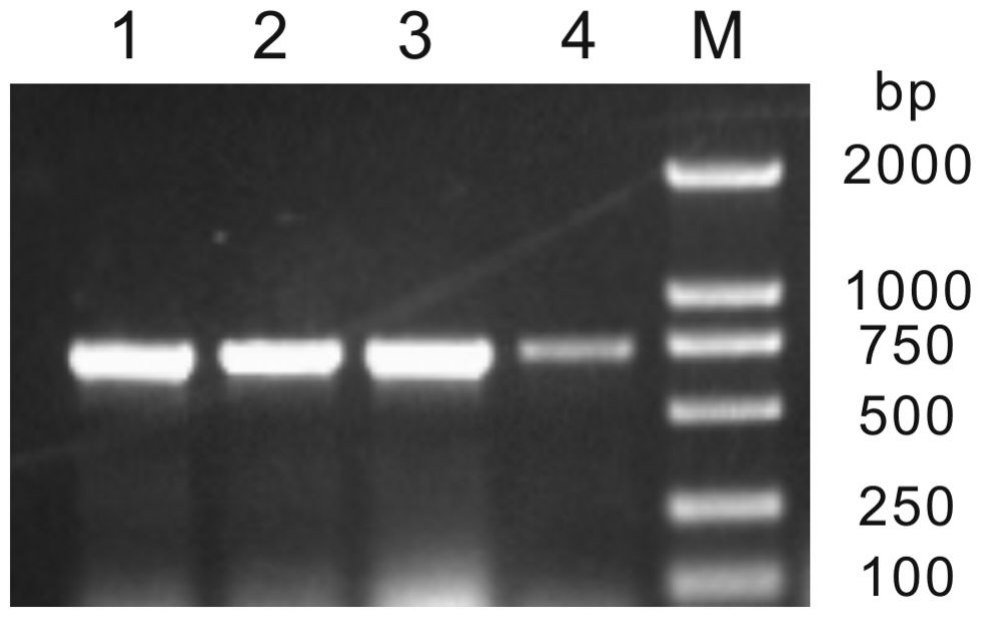
Amplifications of *GbFLA5* ORF from cotton genomic DNA and fiber mRNA. M shows Molecular Marker; lines 1 to 4 shows amplified fragment from Pima 90-53 genomic DNA, CRI 8 genomic DNA, Pima 90-53 fiber mRNA (25 DPA) and CRI 8 fiber mRNA (25 DPA), respectively.

To compare the ORF sequences of *FLA5* both in cDNA and genomic DNA between the Sea Island cotton and the Upland cotton, a primer pair of ORFF and ORFR (Table 1) was used to amplify corresponding ORF sequences from 25 DPA fiber cDNA and genomic DNA. PCR were carried out as described in methods and the results were shown in Figure 3. Amplified products in the four PCR reactions exhibit identical size in length. The bands were cloned, sequenced and analyzed. Interestingly, the sequences in *FLA5* ORF were identical in both Sea Island cotton and Upland cotton, and the cotton *FLA5* is revealed as an intronless gene.

### Amino acid sequence analysis of GbFLA5

From the deduced ORF, the *GbFLA5* encodes a putative polypeptide of 239 amino acids residues in length with a predicted molecular mass of 25.41 kDa and a theoretical isoelectric point of 8.63.

Using the online SMART program, the domain architecture features of the deduced protein of GbFLA5 was identified. The GbFLA5 was predicted to be secreted protein with a predicted signal peptide of 24 amino acids at the N’ terminus, and cleavage site located between Cys24 and Ser25. The region starting from position Thr82 to Leu188 is a typical structure characteristic of a fasciclin-like functional domain (SMART accession number of smart00554) of 107 amino acids. Also, the online program analysis of big-PI Plant Predictor revealed that the GbFLA5 has a C-terminal signal for the addition of a GPI anchor. Therefore, the deduced GbFLA5 protein contains an N-terminal signal sequence, two AGP-like domains, one fasciclin-like domain and a GPI anchor signal sequence.

According to the predicted number of fasciclin-like domains, the number of AGP domains and the presence or absence of a putative GPI anchor signal of the reported FLA proteins in *Arabidopsis* [21], rice [22] and Upland cotton [25], they could be classified into four subgroups. The predicted GbFLA5 protein shares the same characters with subgroup A in the research of Huang et al. [25], by a single fasciclin domain flanked by AGP regions and a C-terminal GPI-anchor signal.

### Phylogenetic analysis and homologous alignment of GbFLA5

**Figure 4:**
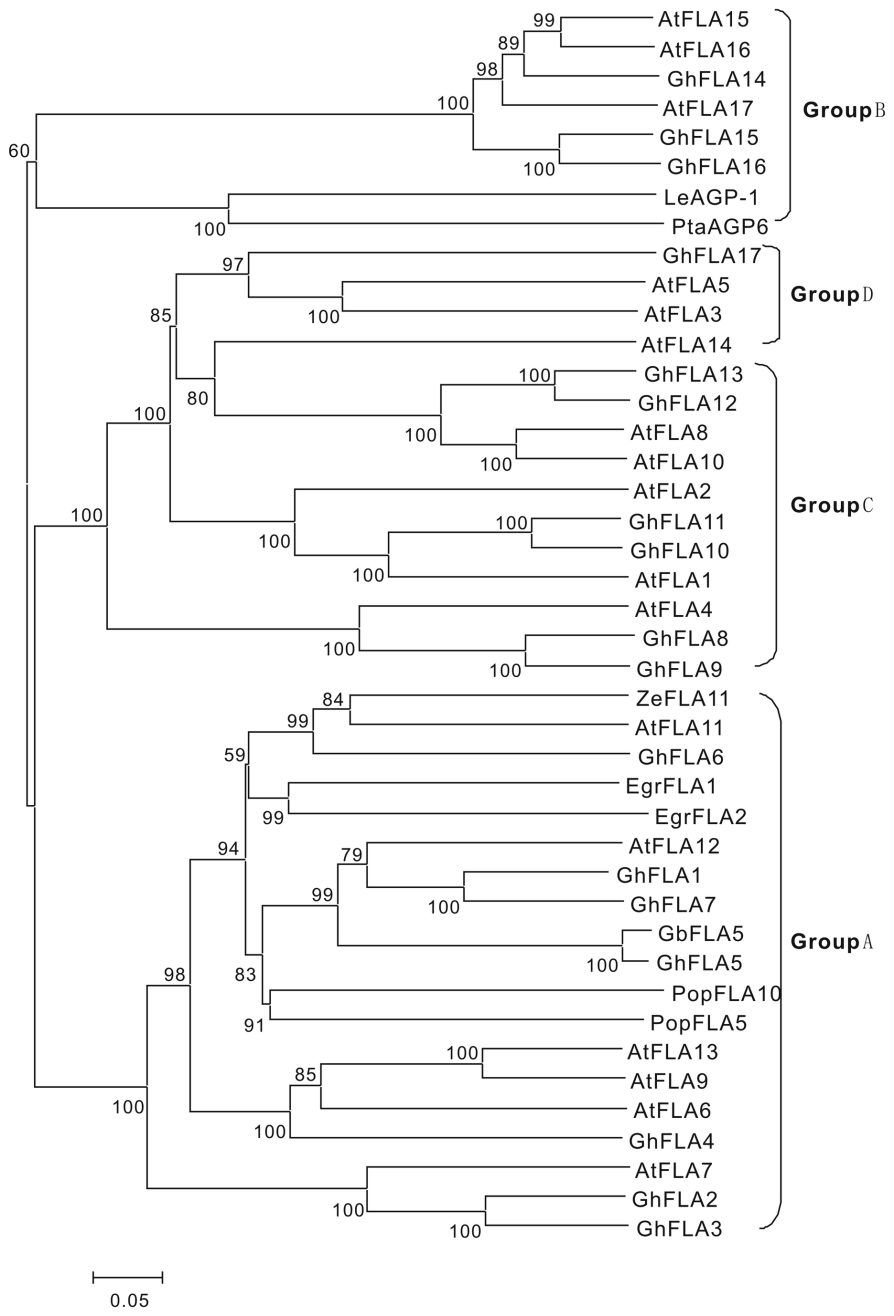
Phylogenetic relationships of GbFLA5 with homologous FLAs in Upland cotton and *Arabidopsis* . The neighbor-jointing tree was constructed with MEGA 4.0. Subgroup of A to D reference to Huang et al. (2008).

To further structurally explore GbFLA5 with other reported plant FLA proteins, the phylogenetic analysis of the deduced GbFLA5 protein with 41 FLAs collected from other plant species was conducted by using Mega 4.0 package. The phylogenetic relationship (Figure 4) shows that GbFLA5 belongs to the subgroup A with other 18 FLA proteins, including GhFLA2, GhFLA5, GhFLA6, AtFLA11 and AtFLA12.

**Figure 5:**
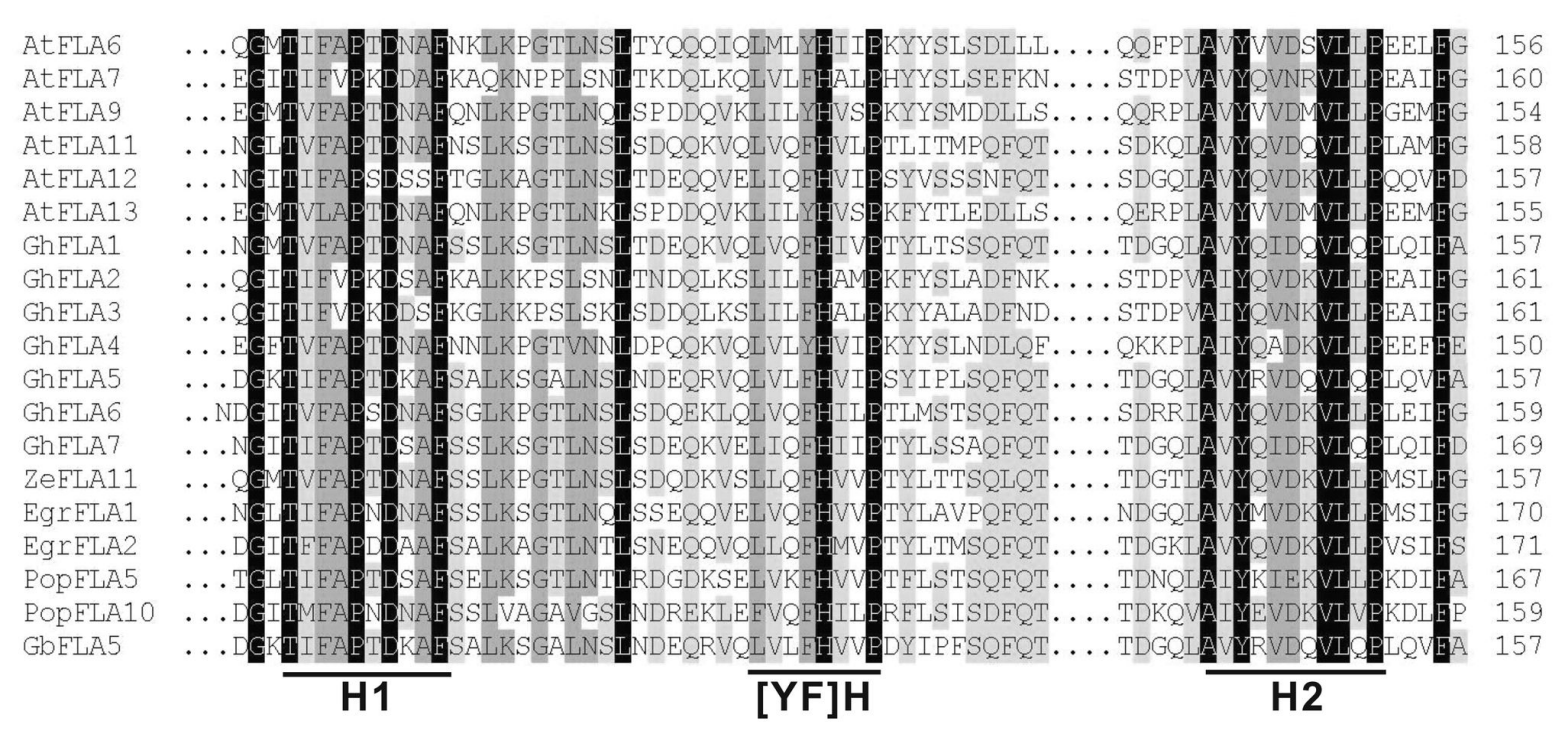
Multiple sequence alignment of the fasciclin-like domain from GbFLA5 and the homologous FLA proteins in plants. The alignment was generated by ClustalW2 program (http://www.ebi.ac.uk/Tools/msa/clustalw2). The three conserved regions of fasciclin domains (H1, H2 and [FY]H) are underlined.

Using the ClustalW2 program, the multiple alignment of the putative fasciclin-like domains identified in GbFLA5 with seven GhFLAs, six AtFLAs and five other plant FLA proteins in subgroup A (Figure 4) was carried out to better understand the structure of the deduced amino acid sequence encoded by *GbFLA5* gene. The results were shown in Figure 5.

It is evident that all proteins mentioned above appear to be rather conserved in structure. Particularly, in the putative fasciclin-like domains identified in these FLAs, three highly conserved regions depicted in the smart00554 motif sequence, designated as H1 and H2 conserved region and Tyr–His motif (located between H1 and H2 regions), exhibited highly similar structure character with subgroup A of FLAs in Upland cotton [25] and rice species [22].

The conserved amino acid residue in Upland FLA proteins [24,25], Thr/T in the H1 region, Tyr/Y or Phe/F -His/H in the [AY] H region, and the H2 region riched in Val/V and Leu/L, were also found conserved in GbFLA5. The two AGP-like domains in GbFLA5 rich in Pro/P, Ala/A and Ser/S residues (see Figure 2 for protein sequence), share identical features with the cell-expansion and wall-thickness related gene of *AtFLA4/SOS5* in *Arabidopsis* [31].

### Semi-quantitative analysis of *GbFLA5* transcript level in different tissues

**Figure 6:**
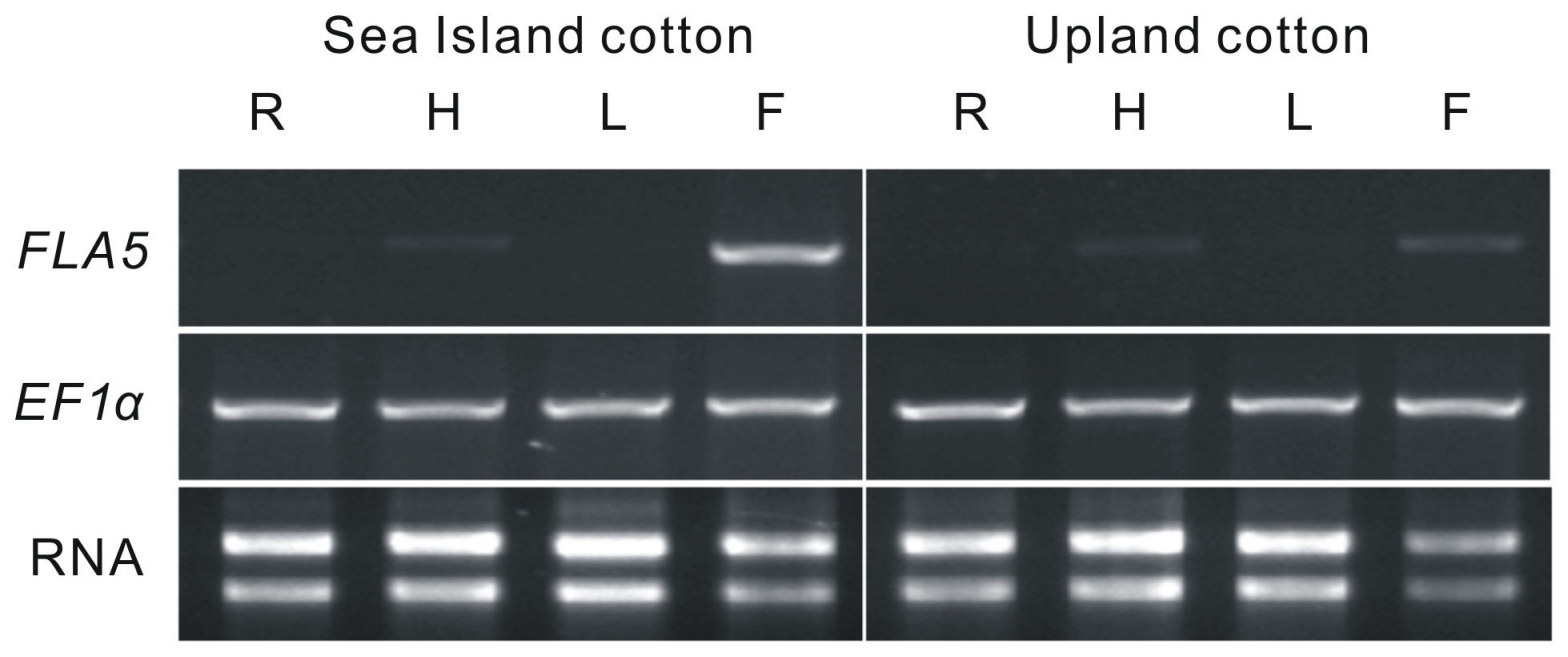
Expression analysis of *FLA5* in various cotton tissues by semi-quantitative RT-PCR. *EF1α* was used as the internal control. Sea Island cotton ( *G* *. barbadense* cv. Pima90-53), Upland cotton（*G*. *hirsutum* cv. CRI 8) and tissues of Root (R), hypocotyl (H), leaf (L) and 25 DPA fibers (F) were marked on the top.

The expression pattern of *FLA5* in various cotton tissues was analyzed by semi-quantitative RT-PCR with specific primers (Table 1). The housekeeping gene of *EF1α* was used as an internal control in the experiment. As shown in Figure 6, transcripts of *FLA5* in the cotton root, hypocotyl and leaf tissues examined showed no apparent differences between the two *Gossypium* species of Sea Island cotton and Upland cotton. Comparing to *EF1α*, the expression level of *FLA5* is significantly lower in hypocotyl, and nearly indiscernible in root and leaf. But the *FLA5* in 25 DPA fibers showed slightly higher expression level in both species, especially in Sea Island cotton fibers. These results implicated that *FLA5* was a typical tissue-specific expressed gene in cotton fibers rather than other plant tissues.

### Relative expression analysis of *GbFLA5* mRNA in developing fibers

**Figure 7:**
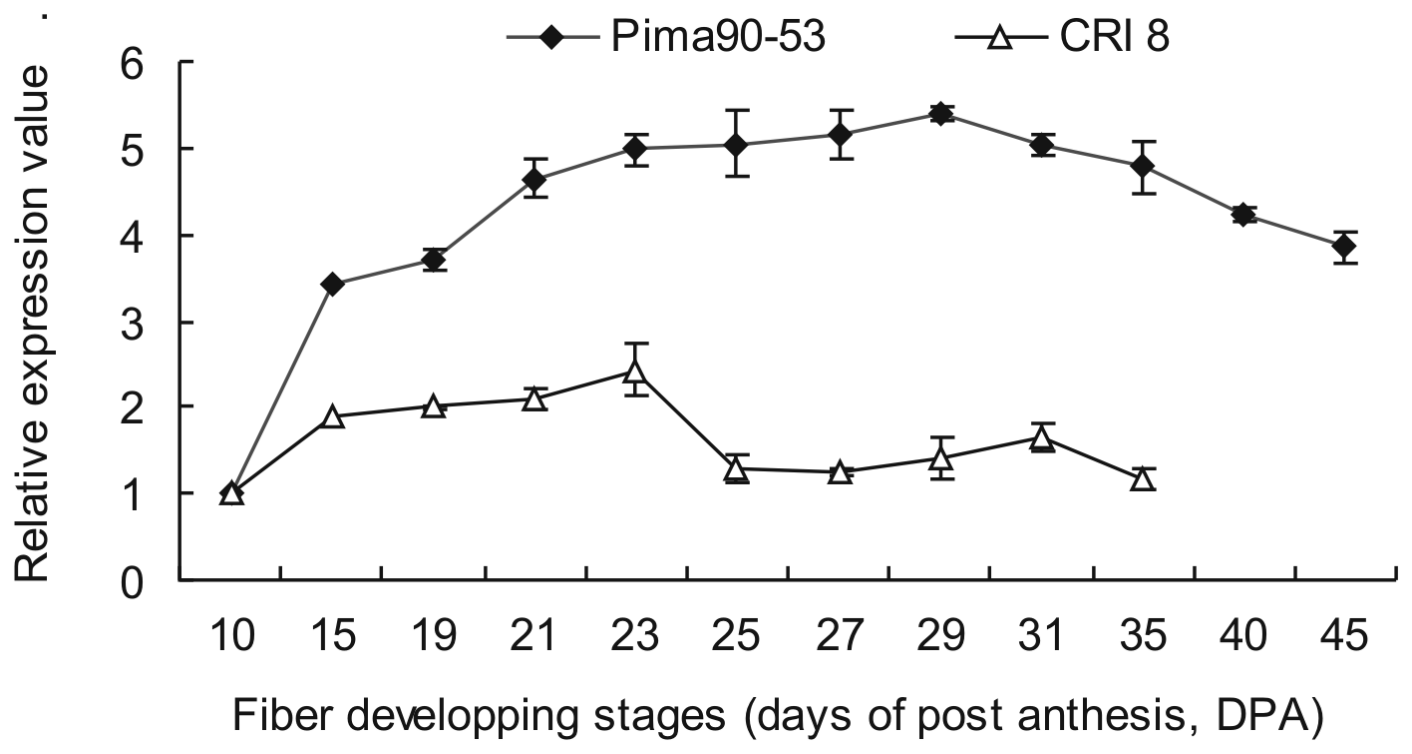
Relative expression analysis of *FLA5* mRNAs in developing fibers by real-time quantitative PCR. *EF1α* was used as the internal control. Cotton fiber development stages (DPA) are as showed under the X-axis.

The fiber *FLA5* mRNA expression level differences between Upland cotton and Sea Island cotton was also measured by real-time RT-PCR using *EF1α* as an internal control. The expression pattern revealed differential transcriptional intensity in the two cotton species fibers. As shown in Figure 7, the *FLA5* expression level was up-regulated from 10 DPA in both *Gossypium* species. *GbFLA5* in Pima 90-53 was up-regulated rapidly along with fiber development stages, and reached the highest point of 29 DPA, and down-regulated from then on until the last examined stage of 45 DPA. While *GhFLA5* in CRI 8 expressing level in fibers exhibited a modest increase until 23 DPA and remained a relatively low transcript activity from 25 to 35 DPA (expression data absence of *GhFLA5* of 35 DPA and 40 DPA was due to its difficulty in extraction of total RNA from Upland cotton fibers in the both stages). It is an interesting observation that the increasing transcript abundance in both species coincided with the SCW deposition stages and the expression profile exhibited a SCW developmental stage-specific pattern.

In the SCW deposition stage from 15 to 45 DPA detected, *GbFLA5* in Sea Island cotton fibers maintains a significantly higher expression level than *GhFLA5* in Upland cotton fibers throughout almost the whole SCW stage examined, especially in fibers of 25 DPA at which the expression level reached its lowest level in Upland cotton. The result is consistent with the results of cDNA-AFLP transcriptome analysis. Another interesting observation is that this expression intensity difference coincided with the strength difference between the two *Gossypium* species.

Together, the overlapping between *GbFLA5* expression patterns and the SCW deposition developmental features suggest that *GbFLA5* expression could be predominantly associated with SCW deposition developmental stage and it acts as a crucial wall-related protein determining the fiber strength differences between the two *Gossypium* species. This will serve as a good hypothesis for the future experiment.

## Discussion

In this study, a fiber transcript encoding a FLA protein was detected differentially expressed between Sea Island cotton and Upland cotton. The corresponding gene, *GbFLA5*, was isolated and characterized in fiber developing stage in Sea Island cotton. Further analysis suggested that *GbFLA5* is a SCW developmental stage-specific expressed gene by encoding a crucial wall protein related to the fiber strength.

Besides the increasing content of cellulose, the SCW of cotton fibers contain small amounts of structural proteins during the thickening stage. AGP is one of the major wall-related proteins widely distributed throughout the plant kingdom. According to the protein backbone features, the AGPs are generally divided into three classes of typical AGPs, chimeric AGPs and hybrid AGPs [32]. FLAs belong to chimeric AGPs, which possess one or two fasciclin domains, one or two AGP-like domains (rich in Pro residues), and presence or absence of glycosyl phosphatidyl inositol (GPI) anchor addition sites [21]. Depending on specific domain combinations, FLAs have been classified into four general subgroups [21,22,25]. GbFLA5 contains two AGP-like domains, a single fasciclin-like domains and a typical C-terminus GPI site, and shares the common structure with GhFLA1/2/3/4/5/6/7 in the same subgroup A in Upland cotton [25].

Many FLA proteins seem to share similar expression patterns associated with plant SCW development. For instance, the *LeAGP-1* is expressed in tomato SCW thickening and lignifying tissues [33]. *PtaAGP6* is abundantly expressed in differentiating xylem in the wood-forming tissues of loblolly pine [8,34]. The phylogenetic analysis of GbFLA5 protein with a set of reported plant FLA proteins were also determined in this study. FLA proteins positioned in subgroup A appear to be conserved not only in their expressions level, but also in their functions in gymnosperms and angiosperms. Existing evidences indicated that the expression of this kind of FLA proteins was conspicuously associated with SCW deposition or coincided with cellulose synthesis in SCW stages. For instance, *AtFLA11* and *AtFLA12* were specifically expressed in *Arabidopsis* sclerenchyma cells [35] and co-regulated with expression of SCW-specific CesA genes [36]. *PopFLA5* and *PopFLA10* transcripts preferentially accumulated in the tension wood differentiating xylem in poplar [27]. The majority of more than 200 of FLA-like ESTs belonging to subgroup A were present in wood-forming tissues [37]. Similar exhibitions were also observed in *Eucalyptus* [38], flax [39] and *Zinnia* *elegans* [26]. Since differentiating plant xylem cells go through four similar stages as that in developing cotton fibers, SCW deposition processing features of xylem mentioned above will provide valuable inference to the developmental genetics of cotton fibers. Additionally, the similar expression pattern of FLAs with a single fasciclin-like domain in plant SCW development suggested a common regulation in affecting cell wall strength.

Mutations in FLA region that led to defective exhibition in SCW development are more reasonable evidences for genetic effects of fasciclin-like domains in SCW thickening process. For example, the salt overly sensitive mutant (sos5) with a single amino acid substitution in the H2 region of the second fasciclin domain of AtFLA4 causes thinner cell walls than the wild type does [31]. Analysis of *FLA3-*RNA interference transgenic *Arabidopsis* indicated that through participating in cellulose deposition, pollen intine formation was affected by the expression of *AtFLA3* [40]. Also in *Arabidopsis* , MacMillan et al. [15] gained a ﬂa11/ﬂa12 T-DNA knockout double mutant in *Arabidopsis* with reduced tensile strength and reduced tensile modulus of elasticity. These evidences suggested a correlation between the specific expression patterns of FLA proteins in subgroup A and SCW thickening process.

In Upland cottons, *GhFLA2* and *GhFLA6* displayed high transcript levels in SCW thickening stage of 20 DPA fibers [25]. Consistent with the evidences in the present study suggested that the fiber-specific expression of *FLA5* is strongly associated with the SCW deposition in both *Gossypium* species. Interestingly enough, the increasing pattern of the *FLA5* transcript level diverged in the two cotton species. The *GbFLA5* transcript level in Sea Island cotton of “Pima90-53” increased from 15 DPA and maintained relatively high level throughout the SCW stage, while its opponent in Upland cotton of “CRI 8” maintained relatively low expression level. Together, these suggested the *FLA5* expression level coincided with the thickening degree of its SCW.

The content of cellulose exceeds 94% in the mature cotton fibers and the fiber properties (maturity, strength and extensibility) are directly dependent on the cellulose content or SCW thickness [41,42]. Thus the deposition of cellulose on SCW gives physical strength to cotton fibers. For instance, the immature fibers of cotton mutant of *imim* have 77.5% less crystalline cellulose than the parental lines which results in 60.2% less single fiber strength [43]. On the contrary, higher amount of cellulose in Sea Island cotton fiber than in Upland cotton fiber [44] is consistent with its superior fiber strength to Upland cotton. In addition to the degree of deposition, the orientation of microfibrils could also be a crucial factor affecting fiber strength [45]. Evidence supporting this opinion comes from a universal observation that high fiber strength correlates with low microfibril angle of the cellulose microfibrils orientation in the loading direction. In *Eucalyptus* , a significant negative correlation between FLA transcript abundance and lowered microfibril angle were observed [38]. MacMillan also found that T-DNA knockout *Arabidopsis* double mutant stems of *Atfla11/12* had decreased tensile strength and stiffness, which is accompanied by reduced cellulose content and an increased cellulose microfibril angle in stem cell walls [15].

FLA proteins are found in intercellular spaces, cell walls, and on plasma membranes where cellulose is continuously being synthesized [46]. The accumulation of cellulose has been observed to accelerate across the fiber developmental stages (15–50 DPA) with a large increase from 15 to 20 DPA [44]. *GbFLA5* expression in our studies exhibited similar increasing feature. In *Arabidopsis* stems, the expression of *AtFLA11* coincided with SCW cellulose synthesis [36]. Also in loblolly pine (*PtaAGP6*) and tomato (*LeAGP-1*), the expressions of relevant *FLA* genes are highly correlated with the deposition of cellulose microfibrils [8]. In *Arabidopsis* and *Eucalyptus* stems, a correlation of *FLAs* expression with microfibril deposition orientation was also observed [15]. These observations suggest that cellulose microfibril is impressed by the presence of FLA proteins in cotton fibers.

Taken together, with the results and the evidences above, we suggested that GbFLA5 is a crucial wall protein that may function on the wall through affecting cellulose synthase, imparting cellulose synthesis and the orientation of cellulose microfibrils on SCW, and the fiber strength was determined as a result.

## Supporting Information

Figure S1 Visualized total fiber RNA of Sea Island cotton cv. **Pima 90-53 ( *G* *. barbadense* ) and Upland cotton cv**. **CRI 8 (*G. hirsutum*) on agarose gel with Ethidium Bromide staining**. (A) Total fiber RNA of Upland cotton cv. CRI 8 (*G. hirsutum*). (B) Total fiber RNA of Sea Island cotton cv. Pima 90-53 ( *G* *. barbadense* ). Lane 1, DL5000 DNA Marker (from the top, 5 000 bp, 3 000 bp, 2 000 bp, 1 500 bp, 1 000 bp, 750 bp, 500 bp, 250 bp and 100 bp) ; Lanes 2–13, fiber developing stages of 10 DPA, 15 DPA, 19 DPA, 21 DPA, 23 DPA, 25 DPA, 27 DPA, 29 DPA, 31 DPA, 35 DPA, 40 DPA and 45 DPA, respectively. The absence of 35 DPA and 40 DPA of Upland cotton was due to its difficulty in extraction total RNA from Upland cotton fibers in two stages.
(TIF)

Figure S2 Melting curves of real-time quantitative PCR analyses of *FLA5* transcripts in fibers of Sea Island cotton cv. Pima 90-53 (* G. barbadense *). Three biologic replicates were indicated by A, B and C. *EF1α* was used as the internal control. The two peaks indicated the PCR products of *FLA5* (left) and *EF1α* (right).
(TIF)

Figure S3 Melting curves of real-time quantitative PCR analyses of *FLA5* transcripts in fibers of Upland cotton cv. CRI 8 (*G. hirsutum*). Three biologic replicates were indicated by A, B and C. *EF1α* was used as the internal control. The two peaks indicated the PCR products of *FLA5* (left) and *EF1α* (right).
(TIF)
