# Supplementary figures and images for: Characterization and Expression Analysis of a Fiber Differentially Expressed Fasciclin-like Arabinogalactan Protein Gene in Sea Island Cotton Fibers
Source: PLoS One. 2013 Jul 17;8(7):e70185. doi: 10.1371/journal.pone.0070185 (PMC3714245; doi:10.1371/journal.pone.0070185)

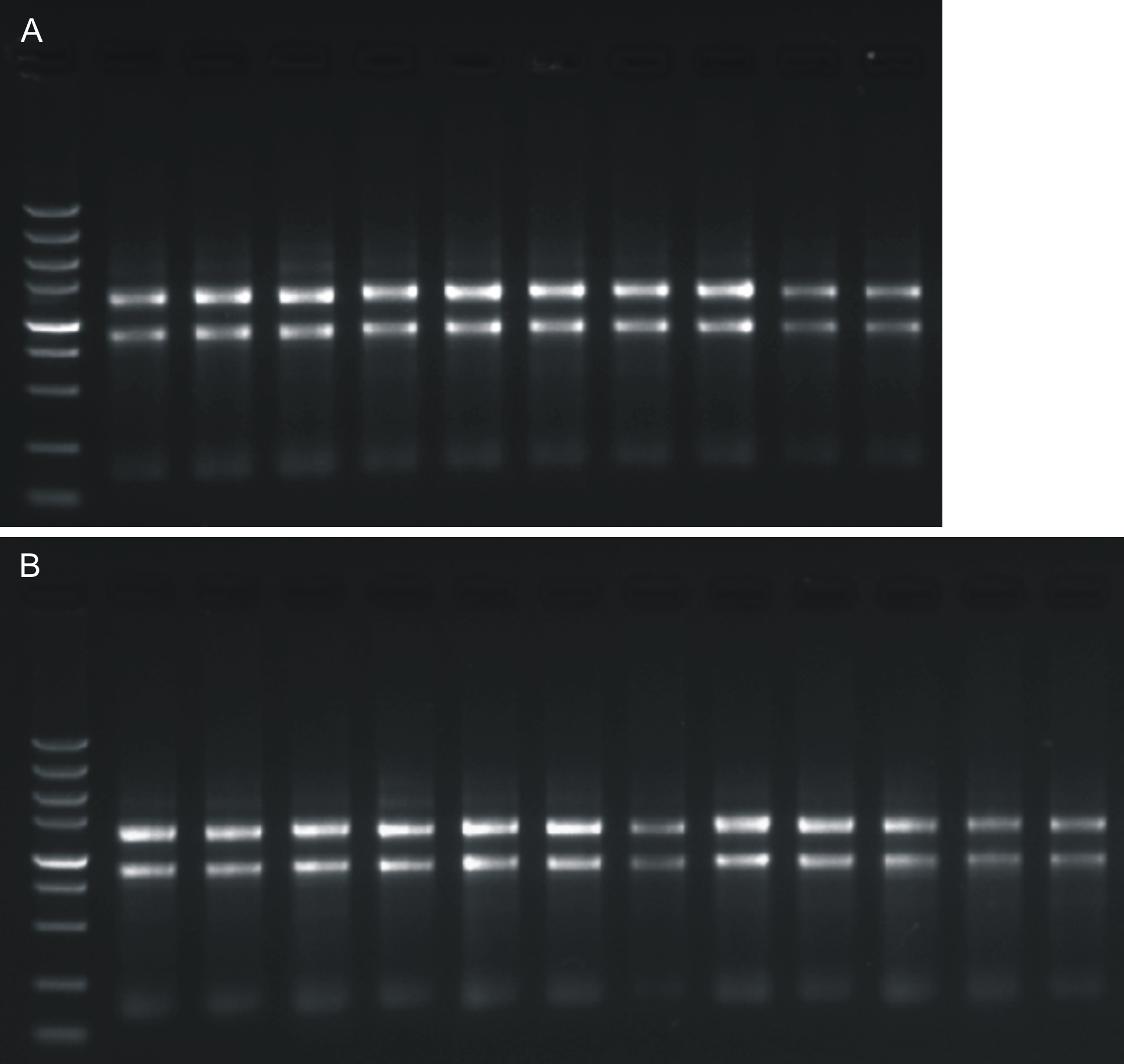

Supplement: Figure S1 — Pima 90-53 ( G . barbadense ) and Upland cotton cv. CRI 8 (G. hirsutum) on agarose gel with Ethidium Bromide staining. (A) Total fiber RNA of Upland cotton cv. CRI 8 (G. hirsutum). (B) Total fiber RNA of Sea Island cotton cv. Pima 90-53 ( G . barbadense ). Lane 1, DL5000 DNA Marker (from the top, 5 000 bp, 3 000 bp, 2 000 bp, 1 500 bp, 1 000 bp, 750 bp, 500 bp, 250 bp and 100 bp) ; Lanes 2–13, fiber developing stages of 10 DPA, 15 DPA, 19 DPA, 21 DPA, 23 DPA, 25 DPA, 27 DPA, 29 DPA, 31 DPA, 35 DPA, 40 DPA and 45 DPA, respectively. The absence of 35 DPA and 40 DPA of Upland cotton was due to its difficulty in extraction total RNA from Upland cotton fibers in two stages. (TIF) [file pone.0070185.s001.tif]

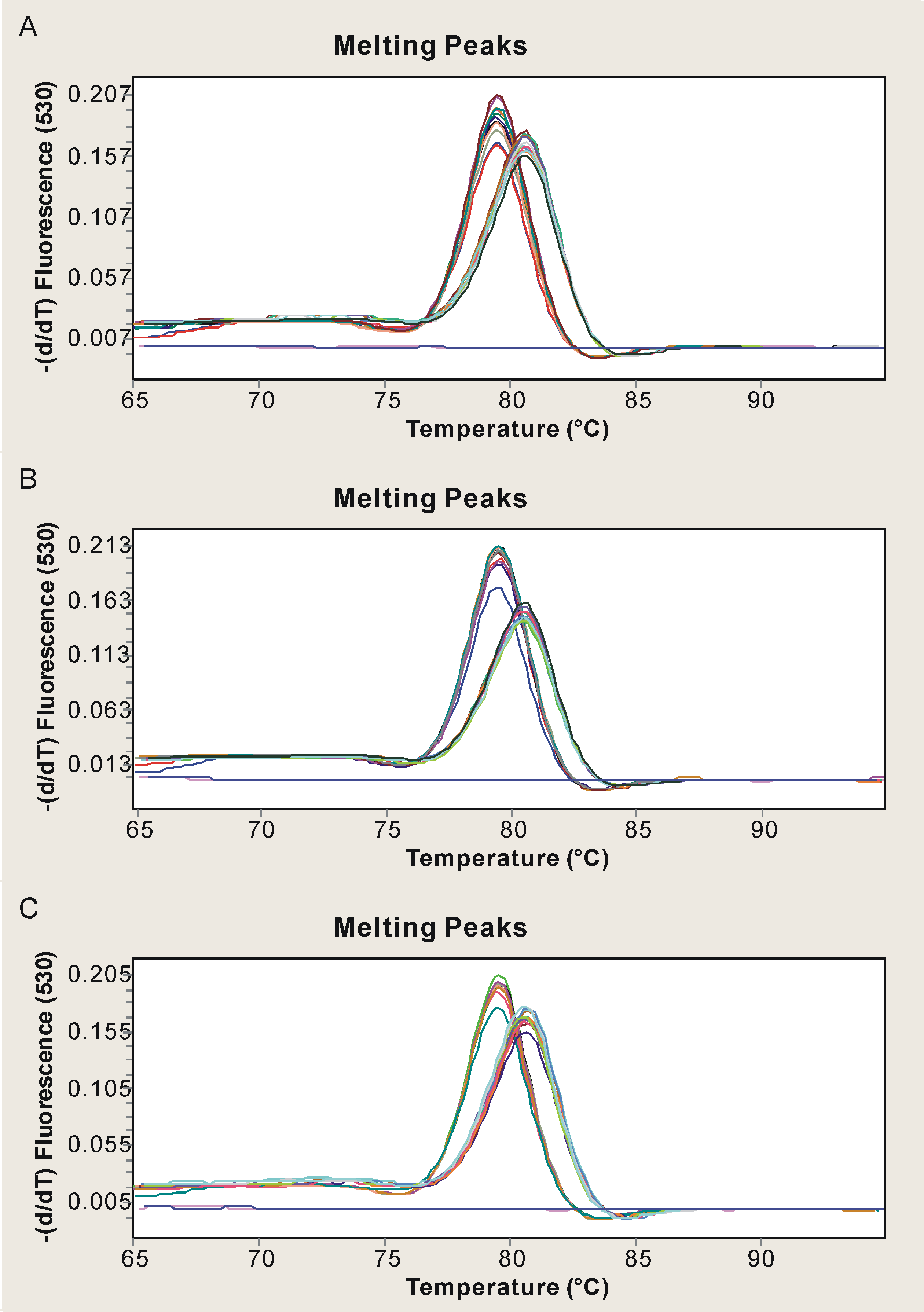

Supplement: Figure S2 — Three biologic replicates were indicated by A, B and C. EF1α was used as the internal control. The two peaks indicated the PCR products of FLA5 (left) and EF1α (right). (TIF) [file pone.0070185.s002.tif]

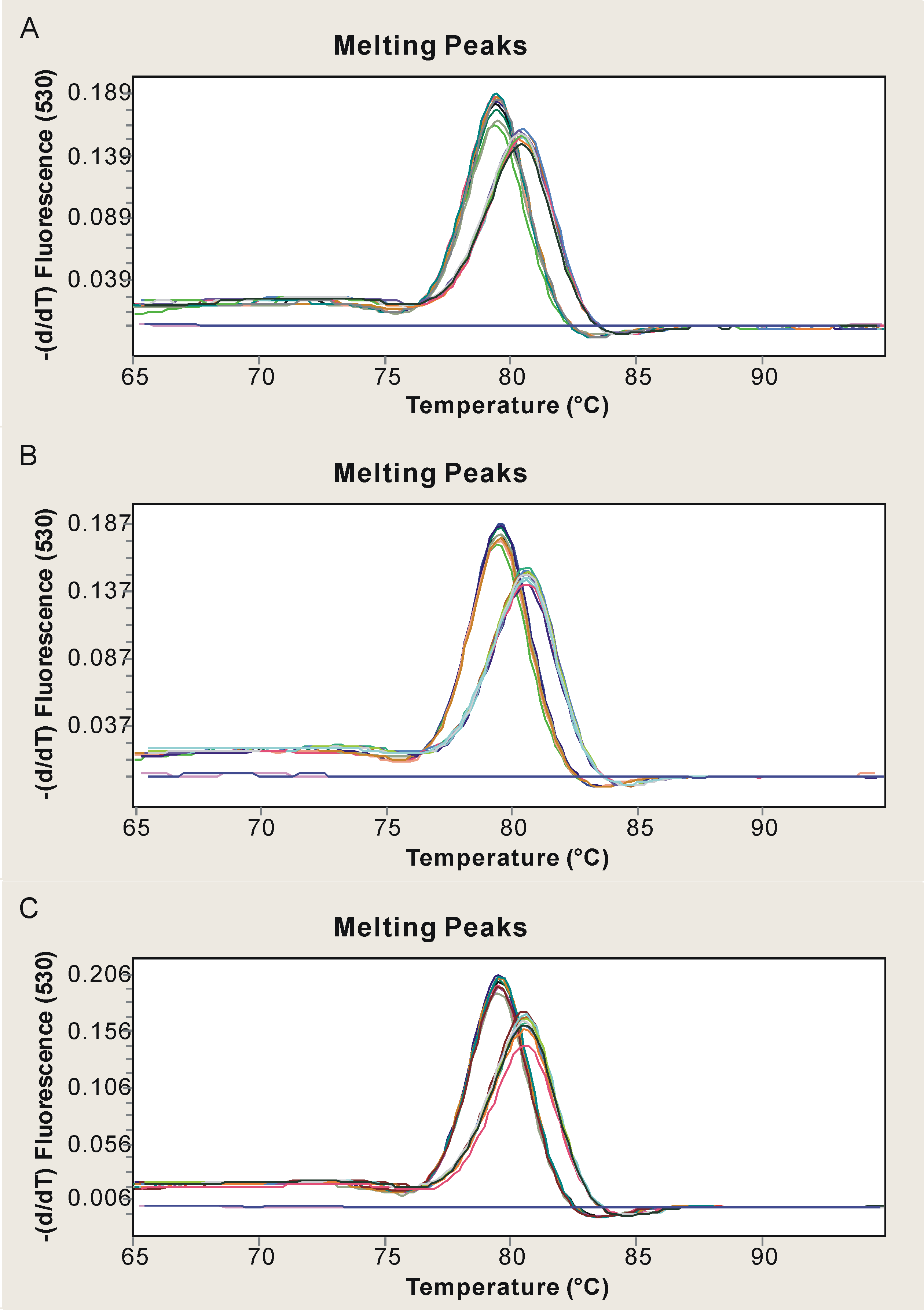

Supplement: Figure S3 — Three biologic replicates were indicated by A, B and C. EF1α was used as the internal control. The two peaks indicated the PCR products of FLA5 (left) and EF1α (right). (TIF) [file pone.0070185.s003.tif]
